# A Comparative Study on the Self-Healing Characterizations and Formulation Optimization of Polyurea Coating

**DOI:** 10.3390/polym14173520

**Published:** 2022-08-27

**Authors:** Xinrui Shen, Zhenyuan Dong, Celine Sim, Yuanzhe Li

**Affiliations:** 1 Department of Natural Sciences, University of Manchester, Manchester M13 9PL, UK; 2 School of Materials Science & Engineering, Nanyang Technological University, Singapore 639798, Singapore; 3 School of Civil, Aerospace and Mechanical Engineering, University of Bristol, Bristol BS8 1QU, UK

**Keywords:** polyurea coating, self-healing, self-healing mechanism, formulation optimization

## Abstract

Self-healing materials, especially self-healing polyurea/polyurethane, to replace traditional coating has been of increasing interest in the past decade. The frequency of regular maintenance work can also be reduced as the coating is capable of forming bonds at ruptured sites. This reduces the cost of maintenance and the risk involved in workers engaging in maintenance work. The extremely short curing time of polyurea coating could potentially outweigh the cost due to its short down time. With a high self-healing efficiency, self-healing polyurea could be the ultimate choice of protective coating. This report aims to find the optimum formulation for fabrication of polyurea with a high self-healing efficiency. This is conducted by changing the composition of the components chosen for formulation of polyurea. The choice of isocyanate and amine is varied to explore its impact on chain mobility and microphase separation, which are important factors affecting self-healing efficiency. A series of characterizations, including ATR-FTIR, DSC, optical microscope and mechanical tester, is used to analyze the factors affecting the self-healing efficiency of fabricated polyurea and to eventually determine the best formulation. The ideal formulation of toluene 2,4 diisocyanate-amine (TDI-P1000) polyurea managed to achieve a self-healing of 42%. Further studies could be done to include multiple healing mechanisms after different area of polyurea to boost its self-healing efficiency after repeated healing.

## 1. Introduction

In the midst of the rising global cost of corrosion, solutions such as application of a protective coating and employing regular maintenance work have been put in place. All of these efforts have been made primarily for the lifetime extension of what is being protected [1]. To further reduce need for regular maintenance, the area of self-healing materials is explored. Polyurethane has always been the more popular coating among other polymers [2] because of its lower cost of raw materials, easy fabrication, and fast curing time [3,4]. Although polyurea is known to cost more, it has an even shorter curing time [5]. The extremely short curing time of polyurea coating could potentially outweigh the cost due to its short down time [6]. With a high self-healing efficiency, self-healing polyurea could be the ultimate choice of protective coating [7]. In this series study, a protective layer of polyurea coating is proposed, which can be prepared quickly and used for a long time with minimum downtime [8]. Quick preparation can ensure fast coating of substrate, while minimum downtime is to maintain constant protection of substrate. Besides that, if microcracks are not healed in time, the healing components on the fracture surface may undergo unwanted side reactions, such as reduction of disulfide bonds or saturation of hydrogen bonds [8]. These side reactions hinder further self-healing processes. Hence, it is important to accelerate the self-healing process and enhance self-healing efficiency. Therefore, the ability of polyurea to self-heal within a short period of time with an acceptable recovery of mechanical strength is important to reduce the extent of corrosion on substrate [9].

This study aims to find the optimum formulation for polyurea to self-heal in a fixed duration of time, even after multiple cuts at the same location regarding its performance in the aspects healing efficiency of strength. This is conducted by exploring a combination of components. The choice of isocyanate is varied to explore how chain mobility affects the self-healing efficiency of polyurea [10]. The choice of amine (in hard segment or soft segment) is varied to explore how microphase separation can affect the self-healing efficiency of polyurea [10,11]. The samples fabricated will undergo a series of characterizations before being cut, which includes ATR-FTIR, DSC, optical analysis, and tensile test. After being cut, the samples will undergo another round of tensile testing to determine self-healing efficiency. The sample with the highest self-healing efficiency after multiple cuts will be deemed the optimum formulation. 

## 2. Materials and Methods

### 2.1. Materials

Poly (propylene glycol) bis (2-aminopropyl ether) average Mn = 2000 (D2000), isophorone diisocyanate (IPDI), toluene 2,4 diisocyanate (TDI), *p*-phenylenediamine (PPD), and chloroform were from Sigma-Aldrich; 4,4-methylenebus (phenyl isocyanate) (MDI) was from DOW Chemical Company; 4,4′-dithiodianiline (DTDA) and bis (4-aminophenyl) sulfone (DDS) were from Tokyo Chemical Industry; Tetrahydrofuran (THF) was from Tedia; and Versalink P1000 was from Evonik Industries. Table S1 shows a compilation of the function and purpose of the included chemicals.

### 2.2. Fabrication of Self-Healing Polyurea

Due to limited resources and failure in fabrication, there was insufficient chemical for the initial plan (Table S2). The experiment was redesigned to vary choice of isocyanate and choice of amine (in hard segments such as chain extenders or soft segments). The characterizations were conducted to analyze chain mobility, microphase separation, and time before contact. The reasons for fabrication failure are explained further in this section. 

Following the formulation in Table 1, respective amounts of the hard segment and the soft segment are accordingly added. Mixing till a homogenous solution was obtained. The final solution was transferred into a mold and volatilized for 24 h. For complete drying, the mold was placed in an oven at 80 °C for 12 h to remove residual solvent.

### 2.3. Attenuated Total Reflectance-Fourier Transform Infrared Spectroscopy Analysis (ATR-FTIR)

All of the ATR-FTIR measurements were performed using a PerkinElmer Frontier spectrometer equipped with the PerkinElmer Universal ATR Accessory. FTIR spectra were collected in the 600 to 4000 cm^−1^ spectral region, with sensitivity of 4 cm^−1^ over 64 scans. A software, PeakFit, was used for curve fitting. Another software Origin was used for application of Gaussian functions. The integral areas of curves were obtained by means of peak resolving [12].

### 2.4. Differential Scanning Calorimetry (DSC) Analysis

Differential Scanning Calorimeter Q10 was used to determine the glass transition temperature of polyurea samples. Q10 has a temperature accuracy of 0.1 and a temperature precision of 0.05. The samples were heated from −90 °C to 230 °C at a heating rate of 20 °C/min under a nitrogen atmosphere (50 mL/min). 

### 2.5. Optical Analysis

To analyze the healing of cracks, a polarizing microscope Olympus BX53 was used. The samples were placed on a glass slide, then on the stage of the microscope. Micrographs were shown on screen and saved. 

### 2.6. Tensile Test

Dumbbell-shaped polyurea specimens were cut out of the sample sheets according to ASTM-D638 standards. The dimension of specimens was 165 mm, 19 mm, 3.2 mm for length, width, and thickness, respectively. An MTS Mechanical Tester Criterion Model 42 was used to perform stress–strain tests at ambient conditions with a crosshead speed of 20 mm/min [8,13].

## 3. Result and Discussion

### 3.1. Fabrication Features and Results

Fabrication of samples labelled from Sample 3 to Sample 6 were successful. Solid samples of polyurea were obtained. However, Samples 1 to 2 did not form a solid polyurea. Prior to this method of fabrication, two other methods have also been tested to produce a sample based on the initial plan (Figure S1a). The main difference in the fabrication methods is the sequence of components being added.

The initial method was to add D2000 dropwise into a respective amount of diisocyanate to form a prepolymer. Then, the chain extender was added to the prepolymer to form polyurea (Figure S2). However, the prepolymer solidified immediately. Addition of a chain extender resulted in a layer of liquid above the initial solid prepolymer. This fabrication failure could be due to an immediate reaction between diamine and isocyanate. The fast reaction means that the chain extender could not be added into the backbone of the polyurea chain [14]. This could be the reason why the red solution of the chain extender remained as a liquid above the solid prepolymer. 

An improved method is planned to include the chain extender into the backbone of polyurea before solidification. D2000 is added to the chain extender and mixed well. This mixture is then added dropwise to a respective amount of diisocyanate. A liquid mixture is obtained. The mixture is then placed in an oven at 80 °C to remove residual solvent. However, the mixture changed to a dark red color and a solid formed in the mixture (Figure S1b). 

This method of fabrication could not guarantee that the chain would turn out similar to that described in Figure S1b as the chain extender and D2000 are both diamines. Both components could react with diisocyanate and lead to formation of the extender-diisocyanate-chain extender polymer chain or D2000-diisocyanate-D2000 [15,16]. The dark red color could be due to oxidation of the mixture at an elevated temperature. The solid formed could be the reaction between the diisocyanate with either D2000 or a chain extender to establish urea bond. At this point, it seems impossible to fabricate a polyurea with all three components: diisocyanate, D2000, and chain extender. Hence, the samples are fabricated without chain extenders [17]. 

A conclusion that can be drawn from all of the fabrication methods is that D2000 may have reacted with air, leading to it not reacting with polyurea. This can be further supported by sample MDI-D2000-PPD from the first method, a layer of solid uneven solid is obtained when the liquid is removed from the mold. This could possibly be due to D2000 reacting with moisture in the air. In the region underneath, the D2000 has yet to react with air and hence is able to react with diisocyanate. On the other hand, the other long chain diamine, P1000, does not react with air as rapidly and allows reaction with diisocyanate. However, it also cures quickly, which prevents the amendment of the polyurea backbone. In order to fabricate polyurea using D2000, a nitrogen atmosphere is needed. Hence, the formulation is changed to those illustrated in Table 1.

### 3.2. ATR-FTIR Analysis

FTIR is capable of providing both qualitative and quantitative analysis of the samples. In terms of a qualitative analysis, FTIR analysis will show the presence of certain functional groups represented by respective wavenumbers. In terms of a quantitative analysis, the concentration of the functional groups can be determined by the peak areas. FTIR analysis has been used in the characterization of polyurea samples to verify that the fabrication has successfully produced polyurea and also to find the concentration of different types of hydrogen bonds present in the samples.

**Figure 1:**
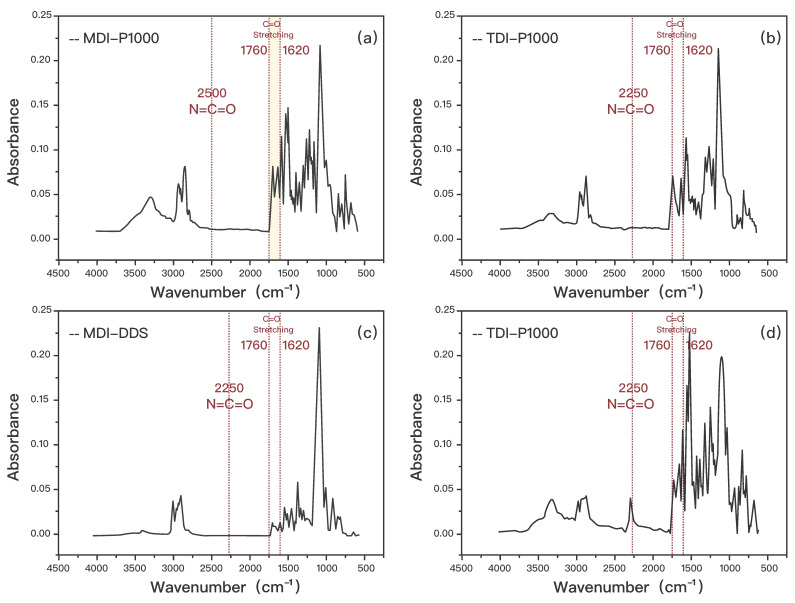
FTIR spectra of (**a**) TDI-P1000, (**b**) MDI-P1000, (**c**) MDI-D2000, and (**d**) MDI-PPD.

To verify that the samples are all polyurea, the peak representing the isocyanate function group at 2250 ${\mathrm{cm}}^{-1}$ should not be present as the urea bond is formed during the reaction of isocyanate and amine functional groups [18]. Besides that, there should also be peaks at approximately 1620–1720 ${\mathrm{cm}}^{-1}$ due to the presence of the stretching vibration of C=O in the urea linkage. There should also be peaks present at approximately 3320–3450$\;{\mathrm{cm}}^{-1}$ due to the presence of the stretching vibration of N-H in the urea linkage [19]. However, the absorption peak of N-H groups is in the same range as the overtone of carbonyl groups [20]. The signals for N-H groups are also weak, hence it is not conducive for quantitative analysis to be conducted. Figure 1 shows the FTIR spectra for the samples. All of the samples have been successfully fabricated to consist of urea linkages.

The purpose of finding the concentration of different types of hydrogen bonds present in the samples is to analyze the packing of the hard segment [21]. The packing of the hard segment plays an important role in self-healing efficiency. With the use of ATR-FTIR analysis, the extent of hard segmental packing can be determined and the formulation’s impact on microphase separation within polyurea can be examined [22]. The peaks at different wavenumbers from 1620 ${\mathrm{cm}}^{-1}$–1760 ${\mathrm{cm}}^{-1}$ represent different types of hydrogen bonds. A compilation of the wavenumber and representative stretching of the carbonyl functional group is shown in Table 2 [23]. 

The presence of “ordered” C=O is representative of a regularly arranged hard segment. A denser packing of the hard segment also means a higher amount of “ordered” C=O is present in the sample [24]. On the other hand, the presence of “disordered” and “free” C=O is representative of an irregularly arranged hard segment [25]. The looser the packing of the hard segment, the higher the amount of “free” and “disordered” C=O. To find the amount of each type of bond, OriginPro software was used to apply a Gaussian function to find the peak area under the graph. Followed by that, a simple law of proportion can be applied to calculate the proportion of each bond and to analyze the overall packing of the hard segment within the function [26].

(1) $$X_{f}=\frac{A_{f}}{A_{f}+A_{d}+A_{o}}$$

(2) $$X_{d}=\frac{A_{d}}{A_{f}+A_{d}+A_{o}}$$

(3) $$X_{o}=\frac{A_{o}}{A_{f}+A_{d}+A_{o}}$$

$X_{f}$, $X_{d}$,$\;X_{o}$ are proportion carbonyl groups with of no hydrogen bond, monodentate hydrogen bonds and bidentate hydrogen bond respectively. $A_{f}$,$\;A_{d}$,$\;A_{o}$ are peak areas on the graphs representing carbonyl groups with no hydrogen bond, monodentate hydrogen bonds, and bidentate hydrogen bond, respectively [26,27].

**Figure 2:**
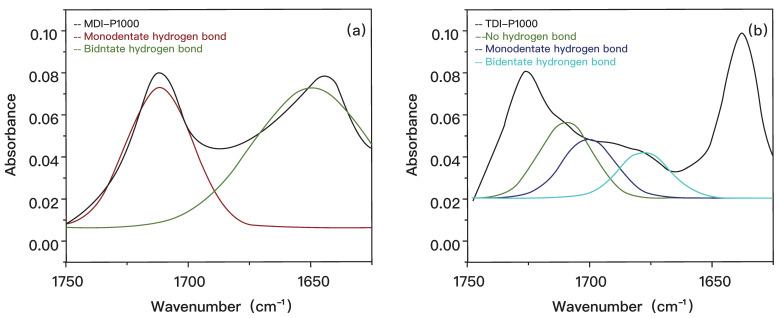
Application of Gaussian function into ATR-FTIR analysis of (**a**) MDI-P1000 and (**b**) TDI-P1000.

Neither MDI-DDS nor MDI-PPD is considered as an ideal formulation group due to its poor transparency and fabrication defects, as indicated in Figure S2. The same FITR characterization has been performed, however, the low absorbance of MDI-DDS and MDI-PPD in the range of 1620 to 1760 cm^−1^ indicates only a low percentage of hydrogen bonding are present in these samples [27]. Self-healing capability in MDI-DDS and MDI-PPD is very limited and hence excluded from the following quantitative analysis. Figure 2 shows the application of a Gaussian function onto the graph using OriginPro software for MDI-P1000 and TDI-P1000 [28].

**Figure 3:**
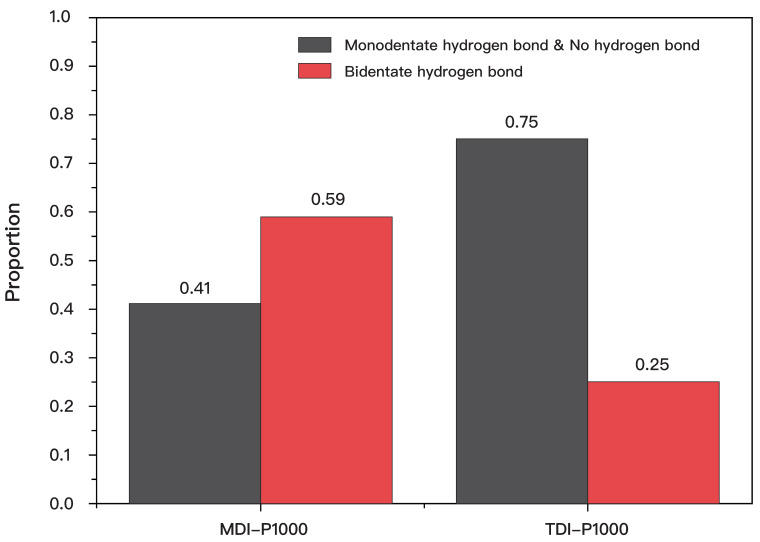
Comparison of bonds in MDI-P1000 and TDI-P1000.

By using Equations (1)–(3), the proportion of carbonyl groups with no hydrogen bond, monodentate hydrogen bonds, and bidentate hydrogen bond are compiled in Table 3. The peak area for no hydrogen and monodentate hydrogen bonds cannot be defined distinctively, hence it is taken as one peak. Similarly, the proportion of carbonyl groups for TDI-P1000 with no hydrogen bond, monodentate hydrogen bonds, and bidentate hydrogen bond are also compiled in the right column. Comparison of different types of hydrogen bonds are represented in a bar graph (Figure 3).

Comparing the composition of different types of hydrogen bonds in MDI-P1000 and TDI-P1000 (Figure 3), MDI-P1000 has a higher proportion of bidentate hydrogen bonds than TDI-P1000. Having a higher proportion of bidentate hydrogen bonds means that there is a hard segment of MDI-P1000 that is more tightly packed. This means that the microphase separation between the hard segment and the soft segment is more distinctive as there is a smaller extent of penetration of the soft segment into the hard segment [22,29]. As a result, MDI-P1000 is more phase-separated [30]. On the other hand, TDI-P1000 has a higher proportion of monodentate hydrogen bonds and no hydrogen bond. The hard segment of TDI-P1000 is more loosely packed, which allows for a larger extent of the soft segment to penetrate into it [20,31]. TDI-P1000 is more phase-mixed than phase-separated. The presence of phase-mixed morphology in TDI-P1000 is further verified by a higher absorbance detected at peaks representing hydrogen bonds in TDI-P1000 than MDI-P1000, signifying more dynamic interactions (H-bonding). From FTIR analysis, it is predicted that TDI-P1000 will show better self-healing capability and efficiency than MDI-P1000 [32].

### 3.3. DSC Analysis

Chain mobility is one of the factors that affects the self-healing efficiency of polyurea. Upon reaching the glass transition temperature, the polyurea change from a solid to a rubbery state [33,34]. The soft segment of polyurea gain a higher chain mobility and hence a higher capability to diffuse to the damaged location, forming new bonds with the broken bonds for self-healing to occur. In order to analyze the chain mobility of the soft segment of polyurea at a different temperature, DSC analysis has been conducted to determine the samples’ glass transition temperatures [35]. Glass transition is an endothermic process that does not occur at one temperature but over a range of temperatures. The temperature in the middle of the region is taken as the glass transition temperature [36]. Since the DSC graph was plotted in an “exo up” setting, the glass transition temperature is expected to be represented in the middle of a decline in heat flow, as shown in Figure S3. 

**Figure 4:**
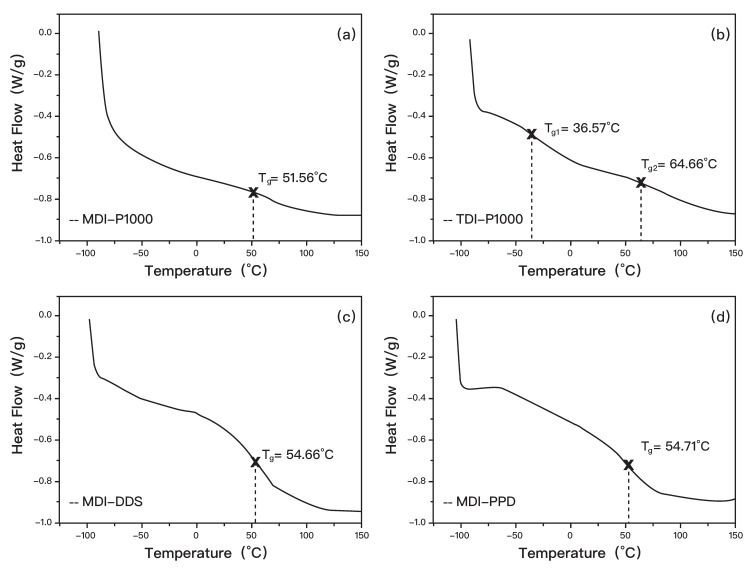
DSC results of (**a**) MDI-P1000, (**b**) MDI-P1000, (**c**) MDI-D2000, and (**d**) MDI-PPD.

From the DSC result of MDI-P1000 (Figure 4), it can be seen that the glass transition temperature is not very distinctive. Upon further rescaling of the graph, the glass transition temperature of MDI-P1000 can be determined as 51.56 °C. The reason for the sample having a non-distinctive wide range of glass transition temperatures could be due to the arrangement of soft and hard segments in the polyurea [37]. According to Lefebvre et al. [38], a broad glass transition temperature is expected for gradient copolymers (Figure S4), where its homopolymers have very different glass transition temperatures. A gradient copolymer is defined as a lamellar with a composition that is smooth, varying such that the domains are never made of purely one homopolymer [39]. In the case of MDI-P1000, the soft segment is comprised of P1000, which has molecular weight of 1000 g/mol. The hard segment is MDI, which has a molecular weight that is approximately five times lower than the soft segment. The significant difference in molecular weight could be representative of a distinct difference in glass transition temperatures between the homopolymers (hard segment and soft segment) [40,41]. 

From the DSC result of TDI-P1000 (Figure 4b), it can be seen that the glass transition temperature is also not distinctive. Similar to MDI-P1000, a molecular weight of TDI is also much lower than that of P1000. Hence, the sample could also be a gradient copolymer. Upon further zooming in at the locations selected, there are two transition temperatures found in the result [42]. One glass transition temperature could be determined as −36.57 °C and the other as 64.66 °C. 

The reason for the appearance of two glass transition temperatures could be due to the presence of two separated phases in the sample. The fabricated polyurea should be a copolymer but not a homopolymer. However, due to the extremely short curing time of polyurea, the hard and soft segments in the sample could not mix well and were unable to form a homogenous solution (copolymer) [43]. Hence, this leads to the observation of two glass transition temperatures, where both copolymer and homopolymer are in the sample. 

Since polyurea that shows capability of self-healing at room temperature have a sub-zero glass transition temperature, the glass transition temperature of copolymer of TDI-P1000 will be taken to −36.75 °C [44]. This temperature will be further verified at a later part of the report, when self-healing capability is tested [45]. From the DSC result of MDI-DDS in Figure 4c, the glass transition temperature can be determined as 54.66 °C, whereas for the DSC result of MDI-PPD, the glass transition temperature can be determined as 54.71 °C (Figure 4d) [46].

From the analysis of DSC results, it can be concluded that the glass transition temperature is more dependent on the diisocyanate (MDI/TDI) used than the amine (P1000/DDS/PPD). All the samples with MDI have glass transition temperatures in the 50 °C range, regardless of the choice of amine. On the other hand, TDI-P1000 has a lower glass transition temperature than samples with MDI. This could mean that, in this case, the chain mobility is more affected by the bulkiness of structure than the molecular weight [47]. 

MDI-P1000 has a higher glass transition temperature than TDI-P1000. This means that the soft segment in MDI-P1000 has a lower chain mobility than the soft segment in TDI-P1000. In another word, a soft segment in MDI-P1000 is more hindered than that of TDI-P1000 [48]. This corroborates with the bulkiness of MDI and TDI structures. Since MDI has a bulkier structure than TDI, the mobility of P1000 in MDI-P1000 is hindered to a greater extent [49]. Hence, a higher energy is needed to allow the polymer chain to move and MDI-P1000 has a higher glass transition temperature. With the information of the glass transition temperature of the samples, if self-healing can take place in these polyurea, it is expected that TDI-P1000 can self-heal at room temperature, while MDI-P1000, MDI-DDS and MDI-PPD will need heating before any potential self-healing can occur [50].

### 3.4. Optical Analysis

The time before surfaces are in contact is one of the factors that affects the self-healing efficiency of polyurea. Once the polyurea has been cut, there has to be contact between two interfaces for self-healing to occur. If no contact is made, there is no chance of self-healing taking place [51]. The waiting time also plays a role in ensuring the occurrence of self-healing instead of self-adhesion. An optical microscope was used to get a magnified view of the cut made on the samples by the razor and to check the self-healing capability of the sample [52]. 

Self-healing capability of the sample is defined as the ability of the sample for self-healing to take place. With the use of microscope, only the capability and speed of self-healing will be analyzed [53]. The efficiency of self-healing in terms of retention of mechanical property will be analyzed in another characterization. For each sample, 3 cm × 1.2 cm rectangular pieces were cut out. On the rectangular pieces, two different types of cuts were made: scratch and clean cut. MDI-DDS and MDI-PPD were too brittle to be scratched or cut by razor. Upon exertion of force, the samples broke into multiple pieces. Hence, MDI-DDS and MDI-PPD were exempted from the scratch test [54].

#### 3.4.1. Optical Analysis for Scratching

A single scratch was made on the samples. The samples were then left at room temperature. The samples were also checked with the naked eye for visible changes at 5 min, 1 h, 3 h, and 6 h. Within the first 6 h, no observable change was seen with naked eyes for all samples. The samples were then placed in an oven at 80 °C. The samples were also checked with the naked eye for visible changes at 5 min, 1 h, 3 h, and 6 h. No observable change was seen with the naked eye for all samples. The samples were then sent under the microscope.

**Figure 5:**
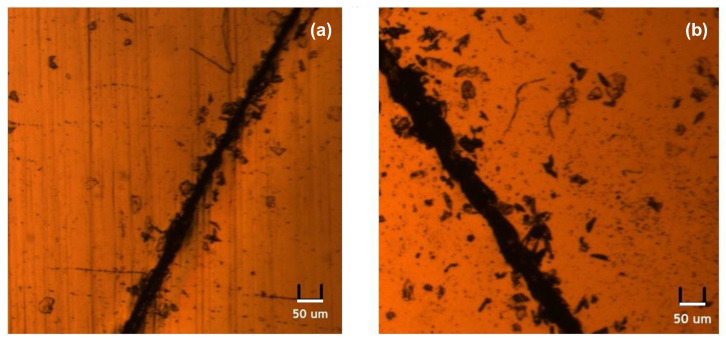
Microscopic images of (**a**) MDI-P1000, (**b**) TDI-P1000.

From the images obtained from the microscope (Figure 5), the razor cut had a width of approximately 50 μm. In both samples, no healing took place. Upon fine tuning, both scratches were still observable on the surfaces of the samples. Hence, MDI-P1000 and TDI-P1000 showed no self-healing capability for scratching. 

#### 3.4.2. Optical Analysis for Cutting

**Figure 6:**
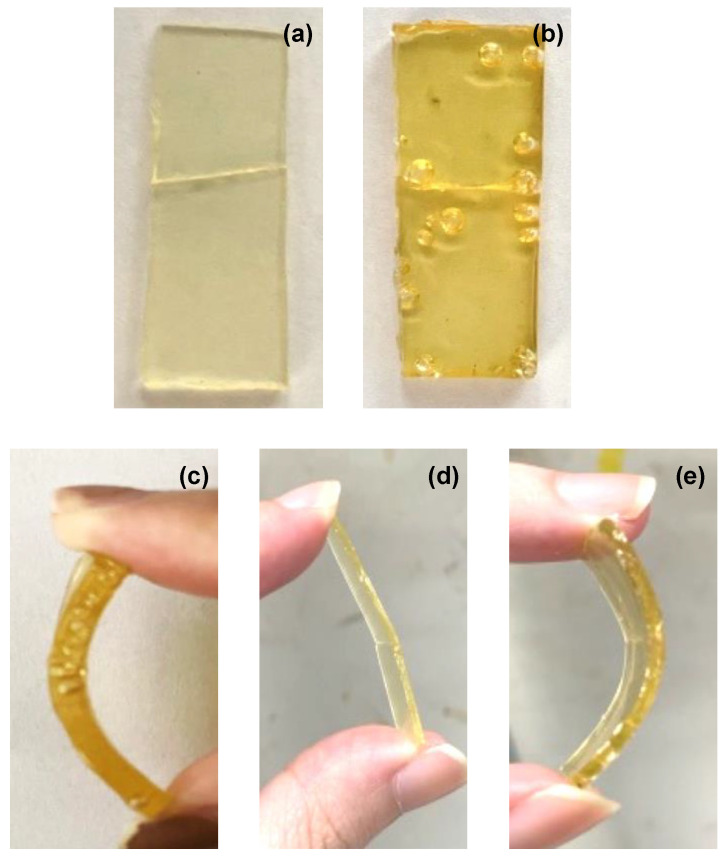
After 6 h self-healing features of (**a**) MDI-P1000, (**b**) TDI-P1000 and bending of (**c**) TDI-P1000 at room temperature, and (**d**) MDI-P1000, (**e**) TDI-P1000 at 80 °C.

The second type of optical analysis is on a rectangular shaped sample cut, which is cut cleanly into two. For this cut, three sets of broken pieces were prepared. The first set (self-healing after 10 min) was placed in contact with each other only after 10 min. For the second (immediate self-healing at room temperature) and the third sets (immediate self-healing at 80 °C), the broken pieces were immediately placed in contact with each other but in different temperature environments. Figure 6 indicates the self-healing features after 6 h of different samples. The difference in curvature does not reflect the degree of softness of the materials, whereas it serves as a demonstration of the cross-section recovery performance of the self-healing progress [55,56].

- Self-healing after 10 min

After cutting the samples into two, the broken pieces were left separately at room temperature for 10 min. After 10 min, the broken pieces were forced together and left at room temperature. Due to MDI-DDS and MDI-PPD being too brittle, it was not possible to properly cut the samples into two. Unevenly shaped and broken pieces of MDI-DDS and MDI-PPD were instead tested [57,58]. 

Broken pieces of MDI-P1000, MDI-DDS, and MDI-PPD were unable to be mended after being left separately for 10 min. The broken pieces of MDI-P1000, MDI-DDS and MDI-PPD remained as separate pieces even after 6 h [59]. On the other hand, TDI-P1000’s broken pieces were able to be attached to each other and be lifted up without falling apart. This means that TDI-P1000 showed self-healing capability [60].

From the results of this set of optical observation, it seems the self-healing results were not coherent with the self-healing capability of samples seen in scratching test. TDI-P1000 showed no self-healing capability in the scratching, but it was shown in such cutting experiments. The reason could be due to the gap between the interfaces. The scratch made in scratching left a 50 μm gap between the two interfaces. However, the broken pieces were forced together in cutting which leaves almost no gap between the interfaces [61]. This shows that the 50 micrometer is beyond the diffusion limit of the samples at room temperature and at 80 °C. 

MDI-P1000 showed self-healing capability, while MDI-DDS and MDI-PPD do not indicates that the presence of long chain diamine is critical in achieving self-healing ability. The presence of long chain diamine allows for the chain to be more flexible than the sample with just a short chain diamine [62]. This explains the brittleness of MDI-DDS and MDI-PPD. The long chain amine could have more donors and acceptors available to form hydrogen bonding, which heals the sample.

- Immediate self-healing at room temperature

For immediate self-healing at room temperature, the broken pieces were immediately forced together and left at room temperature. At 5 min, only TDI-P1000′s broken pieces were connected. After 6 h, the broken pieces of MDI-P1000 and TDI-P1000 managed to form a bridge across the interfaces and stay connected (Figure 6a,b). Nevertheless, the cut at the interfaces were still present for both samples [63]. The gap between the interfaces can still be felt by touch. Slight bending of the MDI-P1000 could cause a fracture along the cut but not on TDI-P1000 (Figure 6c). After 6 h, MDI-DDS and MDI-PPD remained as two separate pieces.

The result from this set shows some similarity with the results obtained from the first set (self-healing after 10 min): MDI-DDS and MDI-PPD do not heal, while TDI-P1000 has self-healing capability. However, the sets differ in whether MDI-P1000 can self-heal. In this set of testing, MDI-P1000 shows self-healing capability. This could be due to the waiting time before the broken pieces were put together [64]. During this waiting time, self-adhesion could not take place because the concentration of open stickers may have dropped further as the open stickers formed looped with dangling chains, and eventually they do not have sufficient open stickers available to form bonds across the interfaces for self-adhesion or self-healing to take place. Putting the broken pieces of MDI-P1000 immediately allow it to form a piece of sample could be due to the process of self-adhesion or self-healing. Considering the low fracture toughness along the cut of the newly combined MDI-P1000 broken pieces, the attachment of broken pieces could be due to self-adhesion or poor self-healing capability.

- Immediate self-healing at 80 °C

For this set of cutting and self-healing experiments, the broken pieces were immediately forced together and left in the oven at 80 °C. The results obtained were similar to those obtained at room temperature. As usual, MDI-DDS and MDI-PPD did not heal. TDI-P1000 managed to heal within 5 min. Although the scar is still visible, the cut could not be felt by touch. The surface of TDI-P1000 after 6 h is smoother than at 5 min. The broken pieces of MDI-P1000 were able to form a single piece within 5 min and slight bending did not result in fracture (Figure 6d,e). Nevertheless, a scar is still visible. In this case, it can be concluded that self-healing occurs for MDI-P1000 instead of self-adhesion. At an elevated temperature of 80 °C, the broken pieces are able to form a stronger bridge across the interface due to the increase in chain mobility [65]. This means that at room temperature, bad self-healing may have taken place instead of self-adhesion as the bridges formed across the interface would not be stronger at a higher temperature of self-adhesion since it is dependent on concentration of open stickers [66]. 

#### 3.4.3. Coherency with FTIR Analysis

From FTIR results, the concentration of hydrogen bonds present in the samples decreased from TDI-P1000, MDI-P1000, MDI-PPD to MDI-DDS. This is coherent with the optical analysis, which showed that at room and elevated temperatures, TDI-P1000 had better self-healing capability than MDI-P1000, while MDI-PPD and MDI-DDS showed no capability. 

#### 3.4.4. Coherency with DSC Analysis

The presence of self-healing capability of TDI-P1000 verified the glass transition temperature of −36.75 °C in DSC analysis. The results obtained from all the sets tested for healing under room temperature are consistent with the conclusion drawn from DSC analysis. In the case where healing is possible, TDI-P1000 with a glass transition temperature lower than room temperature was expected to possess self-healing capability, while the other samples with a glass transition temperature higher than room temperature were not expected to heal at room temperature.

### 3.5. Tensile Analysis

Tensile analysis is used to compare the self-healing efficiency of the samples. Self-healing efficiency is defined as the ratio of the recovered ultimate tensile strength to its initial [67]. It is used to determine the retention of mechanical properties after repetitive healing processes, which is representative of the sample’s self-healing efficiency. To compare the self-healing efficiency, only TDI-P1000 and MDI-P1000 were tested as self-healing capability was seen from optical analysis.

**Figure 7:**
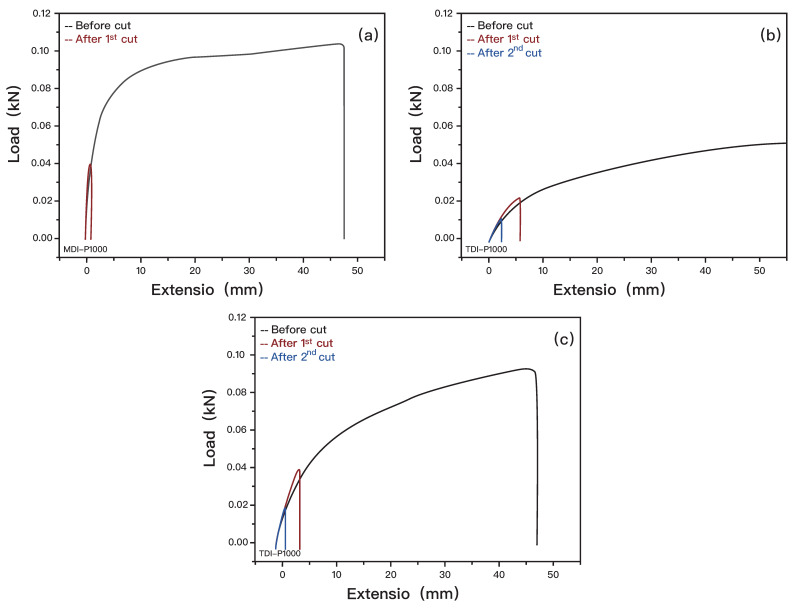
Graph of load vs. extension for before and after cut of (**a**) MDI-P1000, (**b**) TDI-P1000, and (**c**) TDI-P1000.

From the optical analysis, although TDI-P1000 has a smooth surface when healed at 80 °C, the crack can still be seen on the subsurface which indicates that the self-healing may not be 100% complete. During the tensile test, the samples tend to fail along the healing site (Figure 7), which further supported that the samples were not healed fully.

The sample were first tested for tensile strength by pulling till fail. On another sample of the same dimension, a cut that separates the sample into two is made. The second sample is left in the oven at 80 °C to heal for 6 h. The second sample is then tested for tensile strength. The same sample is healed in the oven at 80 °C for 6 h. The sample that is healed for the second time is then tested for tensile strength.

Only two stress–strain curves were obtained as MDI-P1000 could not be healed for the second time. MDI-P1000 has a healing efficiency of 39%, whereas TDI-P1000 has a healing efficiency of 42% from the first heal from the horizontal comparison between Figure 7a,b. It has a healing efficiency of 22% from second heal. From the results obtained from tensile tests, it can be seen that tensile strength decreases with every repetition of breaking and healing. TDI-P1000 has a higher retention of mechanical strength after healing as compared to MDI-P1000. This is coherent with the conclusion derived from FTIR, where a higher number of dynamic interactions (H-bonds) in TDI-P1000 means a higher self-healing efficiency (Figure 7c). This could be the reason for MDI-P1000 not being able to heal the second time, as the self-healing efficiency had degraded [68].

## 4. Conclusions and Position

Self-healing capability and efficiency is highly dependent on the components included during fabrication of polyurea. Important factors that affect self-healing of polyurea include chain mobility, microphase separation, and time before surfaces are in contact. However, there is a trade-off between achieving good chain mobility and adequate microphase separation (phase-mixed). From this experiment, it can be concluded that polyurea with self-healing capability has to be fabricated with isocyanate (hard segment) and long chain amine (soft segment) to achieve the factors mentioned. The choice of isocyanate has more impact on chain mobility than the choice of amine. On the other hand, the choice of amine has more impact on the phase separation than the choice of isocyanate. To achieve the best of both, TDI-P1000 has been tested to portray the best self-healing capability and efficiency of up to 42% at room temperature and elevated temperature even after repetitive healing cycles. 

From the recent available research, self-healing polyurea has mostly been fabricated with D2000. From this experiment, it has been observed that P1000 has a lesser reactivity with air, and it could be a better choice for more commercialized self-healing polyurea.

## Figures and Tables

**Table 1:** Composition of polyurea samples.

Sample Number	Sample Name	Mole of Diisocyanate (mmol)	Mole of D2000/P2000 (mmol)	Mole of PPD/DDS (mmol)	Mole Ratio NH_2_: NCO
1	MD1-D2000	0.5	0.5	-	1:1
2	TDI-D2000	0.5	0.5	-	1:1
3	MDI-P1000	0.5	0.5	-	1:1
4	TDI-P1000	0.5	0.5	-	1:1
5	MDI-DDS	0.5	-	0.5	1:1
6	MDI-PPD	0.5	-	0.5	1:1

**Table 2:** Functional group and their representative wavelength [23].

Wavenumber	Functional Group	Bonding
~1690	C=O stretching of the “free” carbonyl groups	No hydrogen bond
~1670	C=O stretching of “disordered” hydrogen bonded carbonyl group	Monodentate hydrogen bond
~1640	C=O stretching of “ordered” hydrogen bonded carbonyl group	Bidentate hydrogen bond

**Table 3:** Peak area for respective bonds in MDI-P1000.

Bonding	MDI-P1000 Peak Area	TDI-P1000 Peak Area
No hydrogen bond	2.19547	2.23605
Monodentate hydrogen bond		1.81031
Bidentate hydrogen bond	3.06643	1.33266

## Data Availability

The data presented in this study are available on request from the corresponding author.

## Supplementary Materials

The following supporting information can be downloaded at: https://www.mdpi.com/article/10.3390/polym14173520/s1, Figure S1: Reaction to (a) fabricate polyurea according to initial plan and (b) reaction of diisocyanate with amine; Figure S2: (a) Liquid mixture, and (b) After heating in oven, solid formed and liquid mixture turned red; Figure S3: Glass transition temperature portrayed on DSC graph; Figure S4: Gradient copolymer vs block copolymer; Table S1: Names, functions and purpose of materials; Table S2: Initial plan to fabricate polyurea samples.
