# Supplementary material for: A Comparative Study on the Self-Healing Characterizations and Formulation Optimization of Polyurea Coating
Source: Polymers (Basel). 2022 Aug 27;14(17):3520. doi: 10.3390/polym14173520 (PMC9460880; doi:10.3390/polym14173520)
Supplement: Supplementary file 1 [file polymers-14-03520-s001.zip › polymers-1872970-SI.pdf]

polymers

Table S1. Names, functions and purpose of materials.

Chemical name	Full name	Function	Purpose
D2000	Poly(propylene glycol) bis (2-aminopropyl ether)	Long chain diamine (soft segment)	
P1000	Polytetramethyleneoxide-di-p-aminobenzoate	Long chain diamine (soft segment)	
IPDI	Isophorone diisocyanate	Aliphatic diisocyanate (hard segment)	IPDI known good self-healing performance but bad mechanical property
MDI	4,4-methylenebis(phenyl isocyanate)	Aromatic diisocyanate (hard segment)	MDI known for poor self-healing performance but good mechanical property
TDI	Toulene 2,4 diisocyanate	Aromatic diisocyanate	Average self-healing performance and average mechanical property
PPD	p-phenylenediamine	Amine chain extender without disulfide bonds	
DDS	Bis(4-aminophenyl) Sulfone	Amine chain extender without disulfide bonds	DDS has bulkier structure than PPD
DTDA	4,4′-dithiodianiline	Amine chain extender with disulfide bonds	Comparison between different chain extenders.<br>DTDA has similar structure as DDS.<br>PPD has lower Mw than DTDA.
THF	Tetrahydrofuran	Solvent	
$CHCl_3$	Chloroform	Solvent	

Table S2. Initial plan to fabricate polyurea samples.

Sample number	Sample name	Mole of D2000 (mmol)	Mole ratio of IPDI: MDI	Mole of diisocyanate (mmol)	Mole of PPD/DDS/ DTDA	Mole ratio of D2000: diisocyanate: chain extender
1	D2000-IPDI-PPD	0.5	1:0	1.0	0.5	1:2:1
2	D2000-IPDI$_1$ − MDI$_1$ − PPD	0.5	1:1	1.0	0.5	1:2:1
3	D2000-IPDI$_2$ − MDI$_1$ − PPD	0.5	2:1	1.0	0.5	1:2:1
4	D2000-IPDI$_3$ − MDI$_1$ -PPD	0.5	3:1	1.0	0.5	1:2:1
5	D2000-MDI-PPD	0.5	0:1	1.0	0.5	1:2:1
6	(Best of 1-5)-DDS	0.5	-	1.0	0.5	1:2:1
7	(Best of 1-5)-DTDA	0.5	-	1.0	0.5	1:2:1

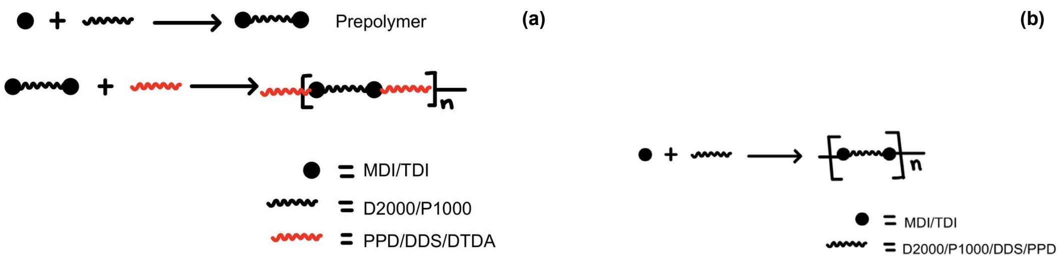

Figure S1. Reaction to (a) fabricate polyurea according to initial plan and (b) reaction of diisocyanate with amine.

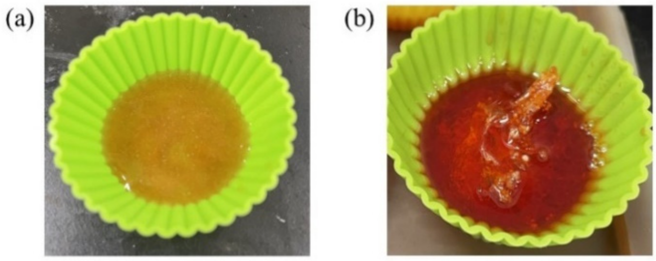

Figure S2. (a) Liquid mixture, and (b) After heating in oven, solid formed and liquid mixture turned red.

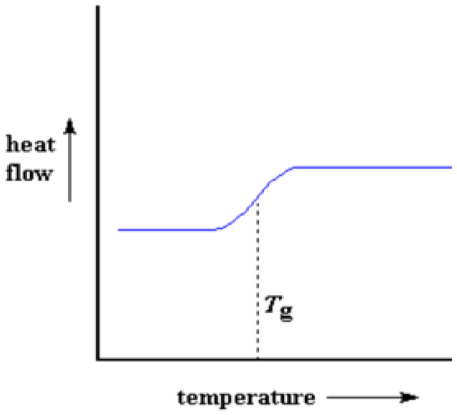

Figure S3. Glass transition temperature portrayed on DSC graph.

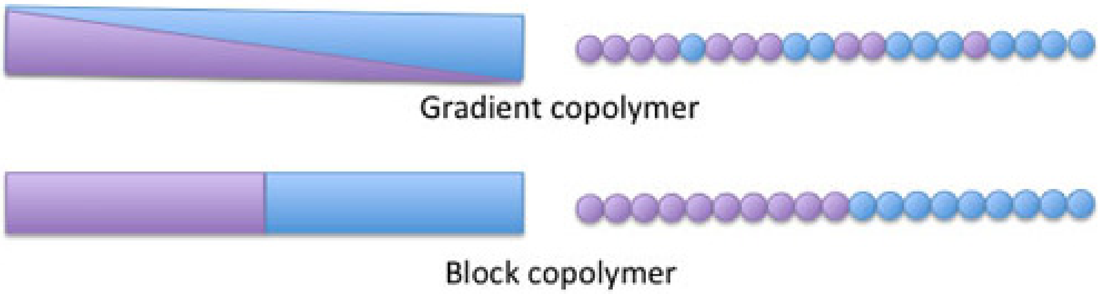

Figure S4. Gradient copolymer vs block copolymer.
